# Supplementary material for: Identifying Genetic Biomarkers Predicting Response to Anti-Vascular Endothelial Growth Factor Injections in Diabetic Macular Edema
Source: Int J Mol Sci. 2022 Apr 6;23(7):4042. doi: 10.3390/ijms23074042 (PMC8999697; doi:10.3390/ijms23074042)
Supplement: Supplementary file 1 [file ijms-23-04042-s001.zip › ijms-1633660-supplementary.pdf]

Table S1. Quality control steps in GenomeStudio (V 2.0) and PLINK (1.9/2.0) for individual array set.

Figure S1: Principal component analysis to detect ethnic outlier.

Figure S2: Scree plot showing the eigenvalues of the first 20 principal components.

Figure S3: Quantile-Quantile plot for association analysis; **A**: Change in central macular thickness; **B**: Change in best-corrected visual acuity.

Figure S4: Regional plots for genome-wide significant locus for change in central macular thickness.

Figure S5: Regional plots for genome-wide significant loci for change in best-corrected visual acuity.

Figure S6A: Manhattan plot for conditional analysis for change in central macular thickness conditioning upon the genome-wide significant lead SNPs.

Figure S6B: Quantile-Quantile plot for conditional analysis for change in central macular thickness conditioning upon the genome-wide significant lead SNPs.

Figure S7A: Manhattan plot for conditional analysis for change in best-corrected visual acuity conditioning upon the genome-wide significant lead SNPs.

Figure S7B: Quantile-Quantile plot for conditional analysis for change in best-corrected visual acuity conditioning upon the genome-wide significant lead SNPs.

Table S2: Baseline and clinical characteristics between responders and non-responders stratified by different categories.

Figure S8: Logistic regression for functional response (Responder: ≥5 ETDRS letters improvement); **A:** Manhattan plot; **B:** Quantile-Quantile plot

Figure S9: Logistic regression for functional response (Responder: ≥15 ETDRS letters improvement); **A:** Manhattan plot; **B:** Quantile-Quantile plot

Figure S10: Logistic regression for functional response (Non-responder: ≥5 ETDRS loss); **A:** Manhattan plot; **B:** Quantile-Quantile plot

Figure S11: Logistic regression for anatomical response (Responder: ≥10% reduction in central macular thickness); **A:** Manhattan plot; **B:** Quantile-Quantile plot

Table S3: Expression data of the potential candidate genes for different eye tissues retrieved from the Ocular Tissue Database (OTDB).

Table S1: Quality control (QC) steps in GenomeStudio (V 2.0) and PLINK (1.9/2.0) for individual array set								
**Array version**	**Starting numbers**	**Post Genome Studio QC numbers**	**Post individual QC (PLINK)**	**Duplicate, non-biallelic, indel SNPs removed**	**Number of SNPs common to all versions of the array**	**After merge**	**Post merge QC (PLINK)**	**Post-imputation QC (PLINK)**
Data-A (GSAMD-24v3-0-EA_A1)	730059 SNPs<br><br>96 individuals	480728 SNPs<br><br>96 individuals	458089 SNPs<br><br>95 individuals<br><br>**QC parameters:**<br>--geno 0.02 (158 SNPs removed)<br>--mind 0.02 (0 individuals removed)<br>--sex (0 individuals removed)<br>--het<br>(1 individual removed)	452328 SNPs<br><br>(5761 SNPs removed)	377657 SNPs<br><br>95 individuals	339682 SNPs<br><br>226 individuals (3 duplicate individuals and 1 ineligible patient)<br><br>Genotyping rate 99.99%	281,952 SNPs<br><br>221 individuals, Genotyping rate=99.99%)<br><br>**QC parameters:**<br>--geno 0.02 (all pass)<br>--mind 0.02 (all pass)<br>--maf 0.01 (57730 SNPs removed)<br>-cryptic related (pi-hat ≥0.1875 =none)	" info R2<0.8" 8,348,433 SNPs remaining out of 39,127,678<br><br>221 individuals, Genotyping rate=95.30%<br><br>**Post QC:** SNPs=2,581,674<br><br>220 samples Genotyping rate=99.30 %<br><br>**QC parameters:**<br>--geno 0.03 (3,122,034 SNPs removed)<br>--mind 0.03 (0 individual removed)

Data-B (GSA-24v1-0_C1)	618540 SNPs<br><br>87 individuals	516336 SNPs<br><br>87 individuals	503869 SNPs<br><br>85 individuals<br><br>**QC parameters:**<br>--geno 0.02 (1072 SNPs removed)<br>--mind 0.02 (0 individuals removed)<br>--sex (1 individual removed)<br>--het (6 SD) (1 individual removed	500209 SNPs<br><br>(3660 SNPs removed)	377657 SNPs<br><br>85 individuals		--het (1 individual removed)<br>PCA- 4 ethnic outliers removed	--maf 0.02 (2,644,725 SNPs removed)<br>--hwe 1E-06<br>--cryptic relatedness (king-cut off ≥ 0.0884, 0 removed)<br>--het (6SD, 1 individual removed)
Data-C (GSA-24v3-0-A1)	654027 SNPs<br><br>51 individuals	637311 SNPs<br><br>50 individuals (1 individual removed due to low call rate)	636715 SNPs<br><br>50 individuals<br><br>**QC parameters:**<br>--geno 0.02 (596 SNPs removed)	624137 SNPs<br><br>(12578 SNPs removed)	377657 SNPs<br><br>50 individuals			

			--mind 0.02 (0 individual removed)<br>--sex (0 individuals removed)<br>-- het (0 individuals removed)					

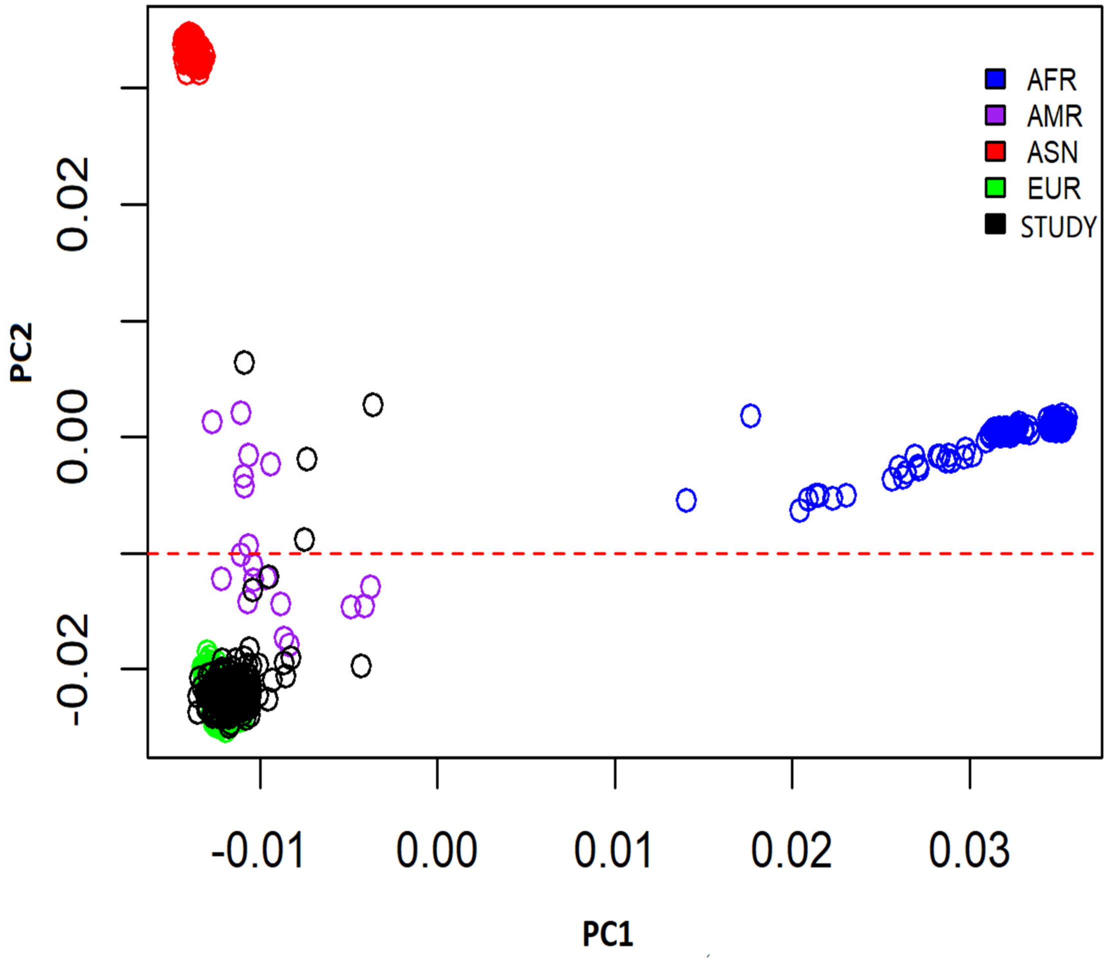

Figure S1: Principal component analysis to detect ethnic outliers.

The x-axis represents the first principal component (PC1) and the y-axis is the second principal component (PC2). Each point represents a single individual distinguished by population origin (AFR=African, AMR= Ad Mixed American, ASN=Asian, EUR = European, STUDY=study cohort). Individuals with a second principal component score less than -0.01 (horizontal line) were excluded from analysis.

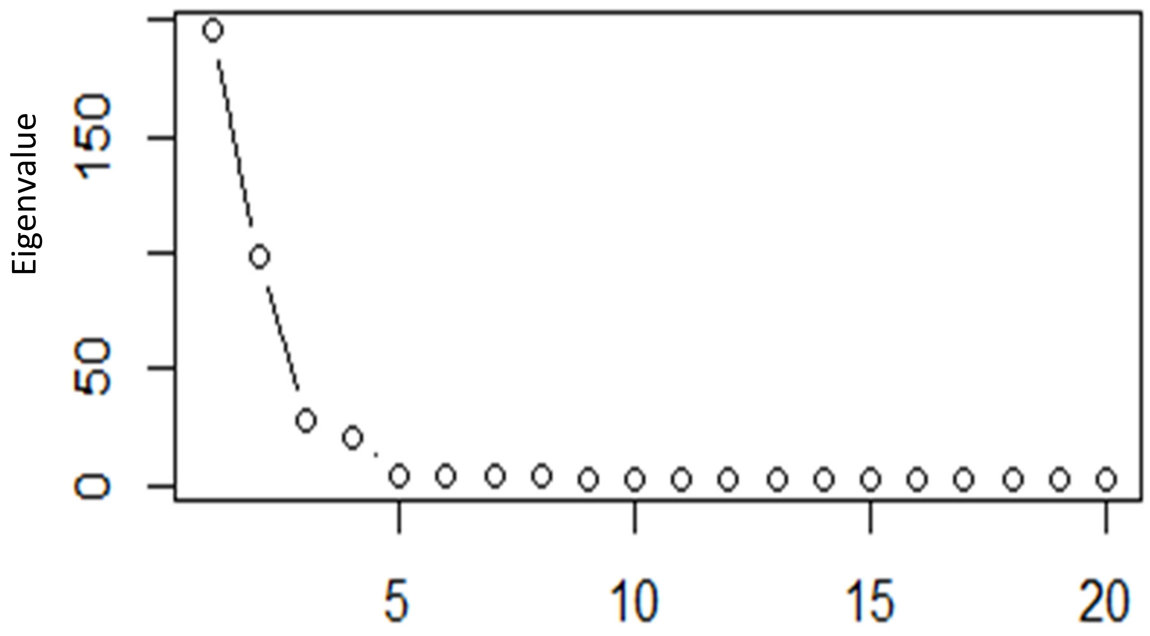

Figure S2: Scree plot showing the eigenvalues of the first 20 principal components (PCs). This plot indicates that the first three principal components explain the majority of the variability in this dataset.

A.

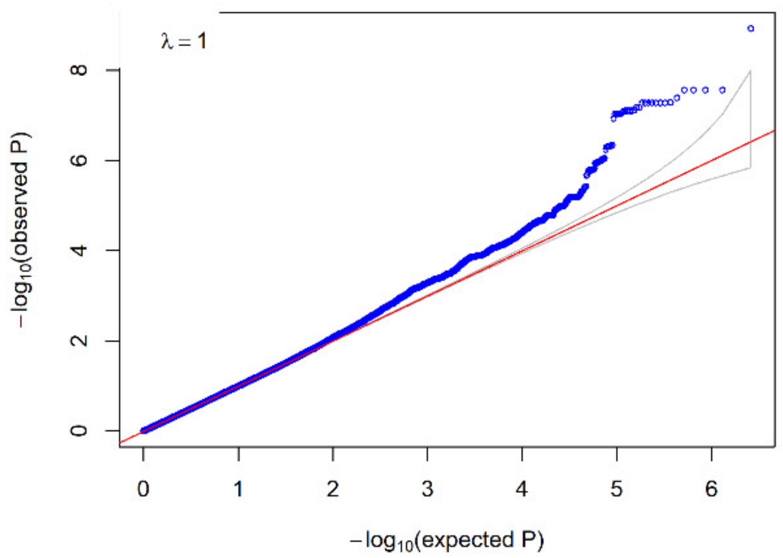

B.

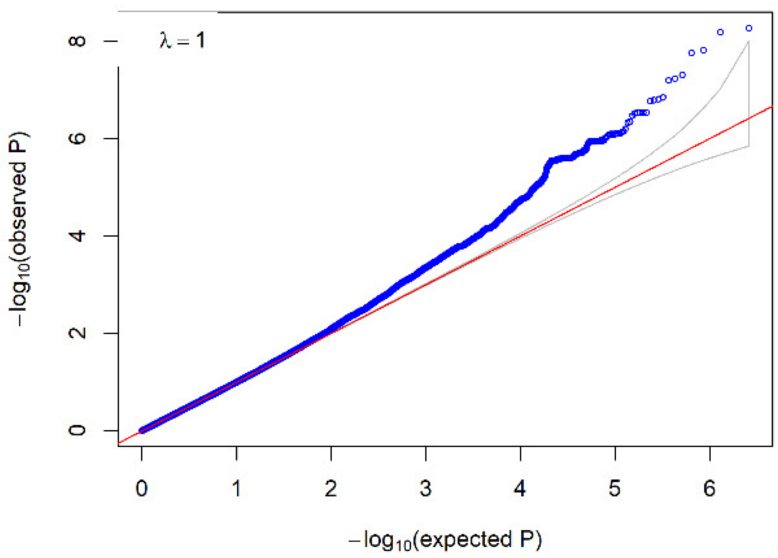

Figure S3: Quantile-Quantile plot for association analysis; **A:** Change in central macular thickness; **B:** Change in best-corrected visual acuity. The plots show the expected −log10 p-values under the null hypothesis on the x-axis, and the observed −log10 p-values on the y-axis adjusting for covariates (λ = 1.000). The red lines represent x=y, and the grey area represents the 95% confidence interval. The λ is a measure of the genomic inflation factor.

A.

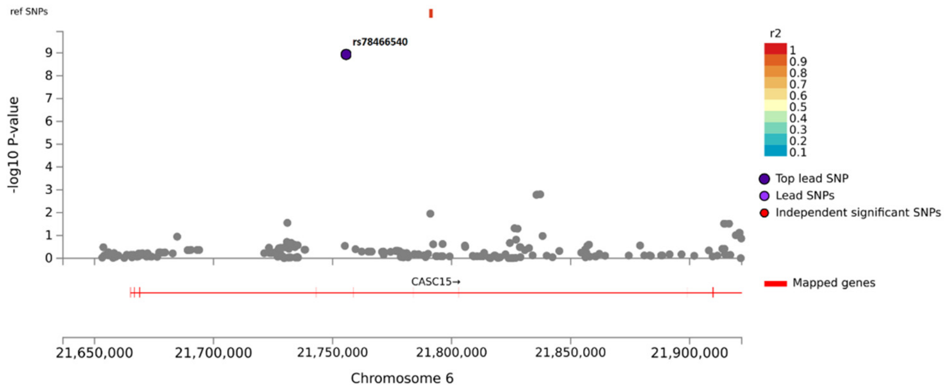

B.

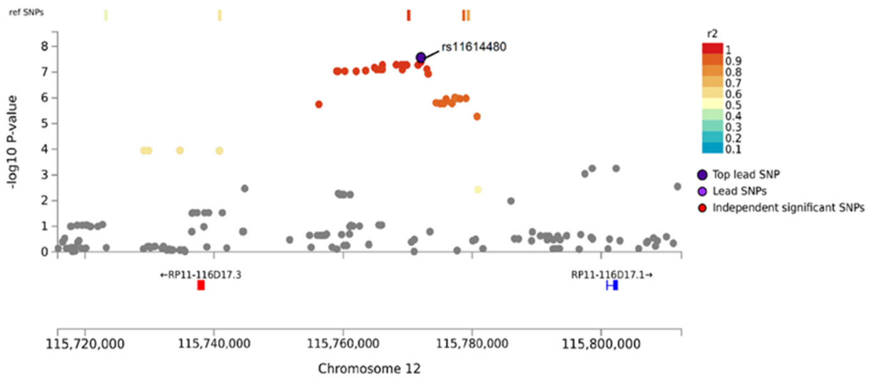

Figure S4: Regional plot for genome-wide significant loci for change in central macular thickness. SNPs are color-coded based on r2 if ≥0.4. Non-GWAS-tagged SNPs in the 1000G (Phase3 EUR) reference genome are shown at the top of the plot as rectangles since they do not have a P-value from the GWAS, but they are in linkage disequilibrium with the lead SNP. SNPs below r2<0.4 are colored grey.

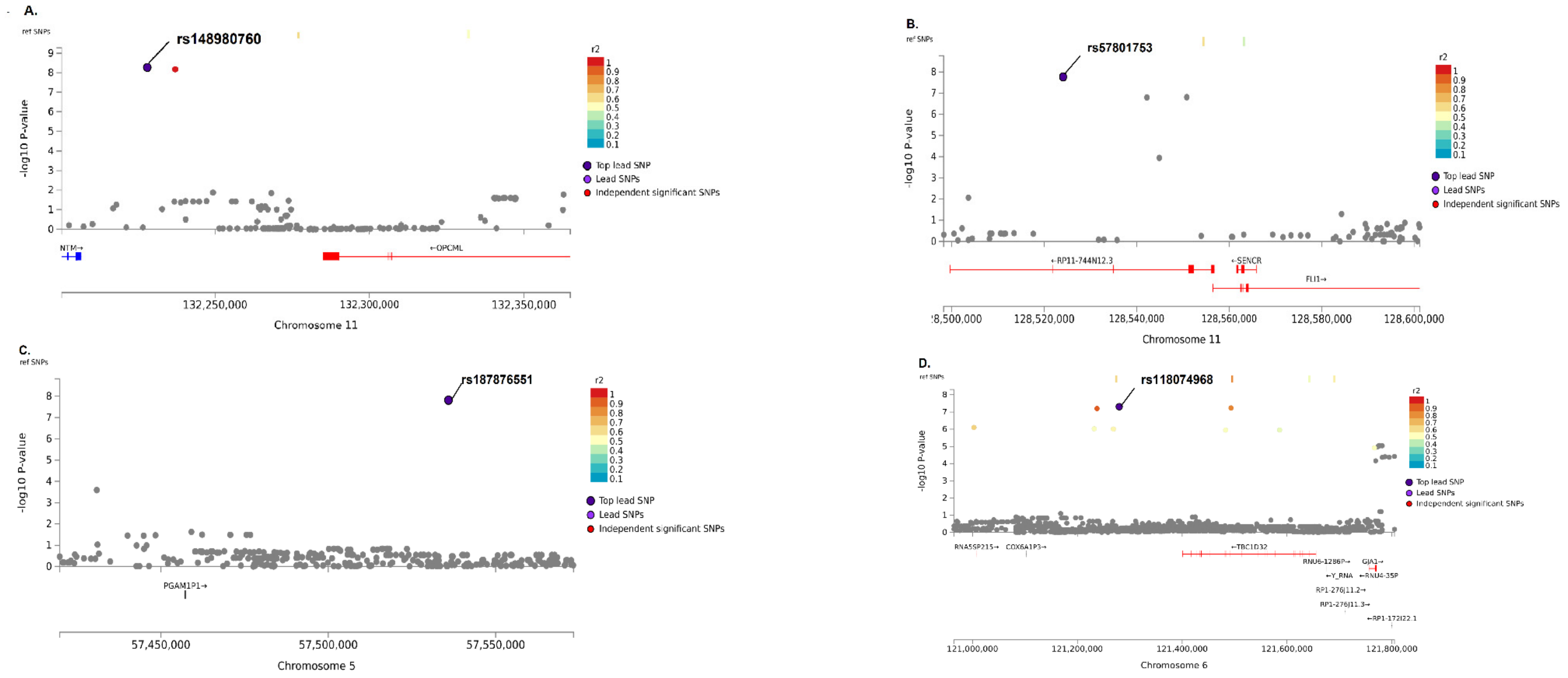

Figure S5: Regional plots for genome-wide significant loci for change in best-corrected visual acuity. SNPs are color-coded based on r2 if ≥0.4. Non-GWAS-tagged SNPs in the 1000G (Phase3 EUR) reference genome are shown at the top of the plot as rectangles since they do not have a P-value from the GWAS, but they are in linkage disequilibrium with the lead SNP. SNPs below r2<0.4 are colored grey.

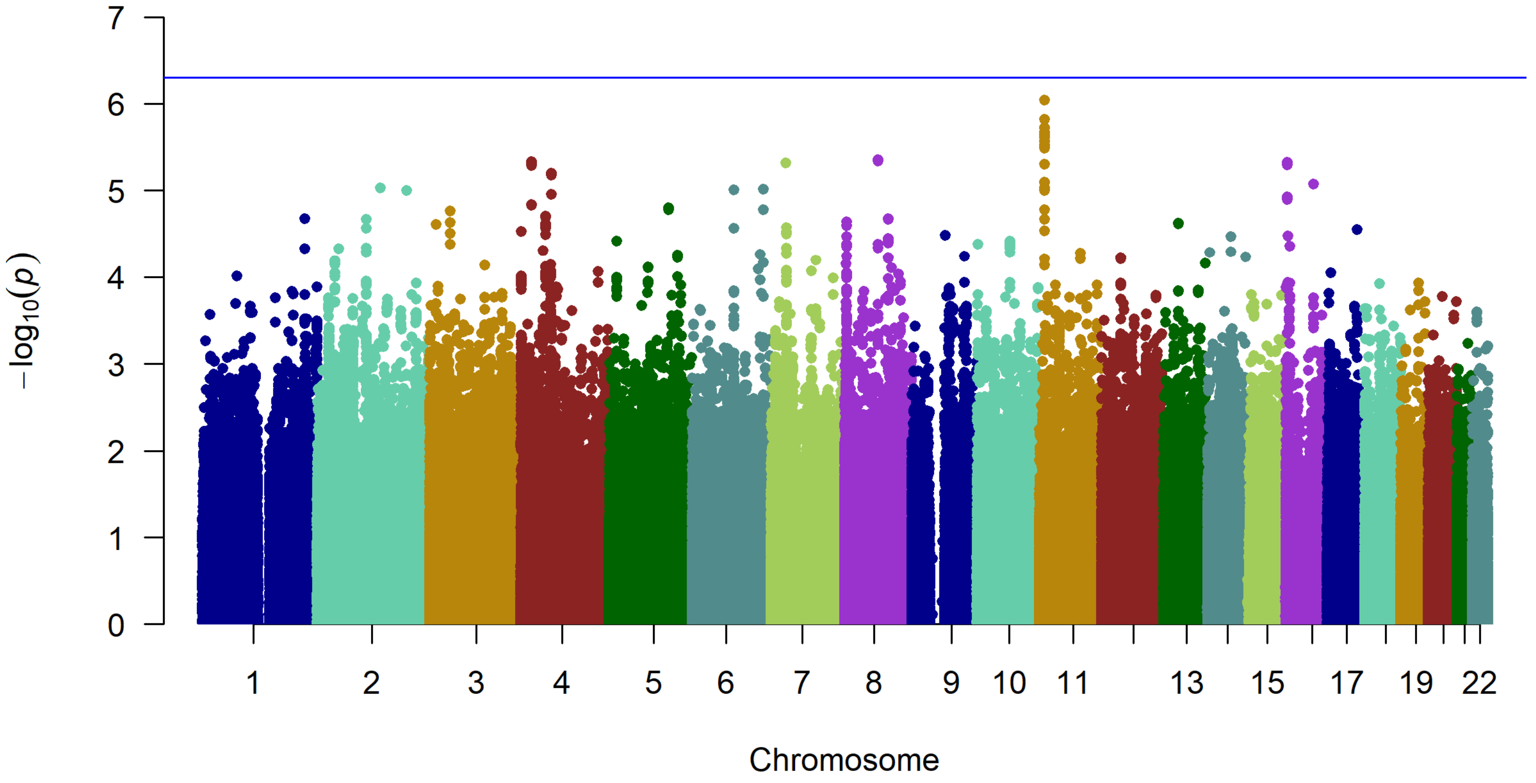

Figure S6A: Manhattan plot for conditional analysis for change in central macular thickness conditioning upon the genome-wide significant lead SNPs (rs78466540, rs11614480). The x-axis represents chromosomal position, and the y-axis represents the −log10 (P-value) of association for each SNP tested. The blue horizontal line corresponds to the threshold for genome-wide suggestive association (P≤5E-07).

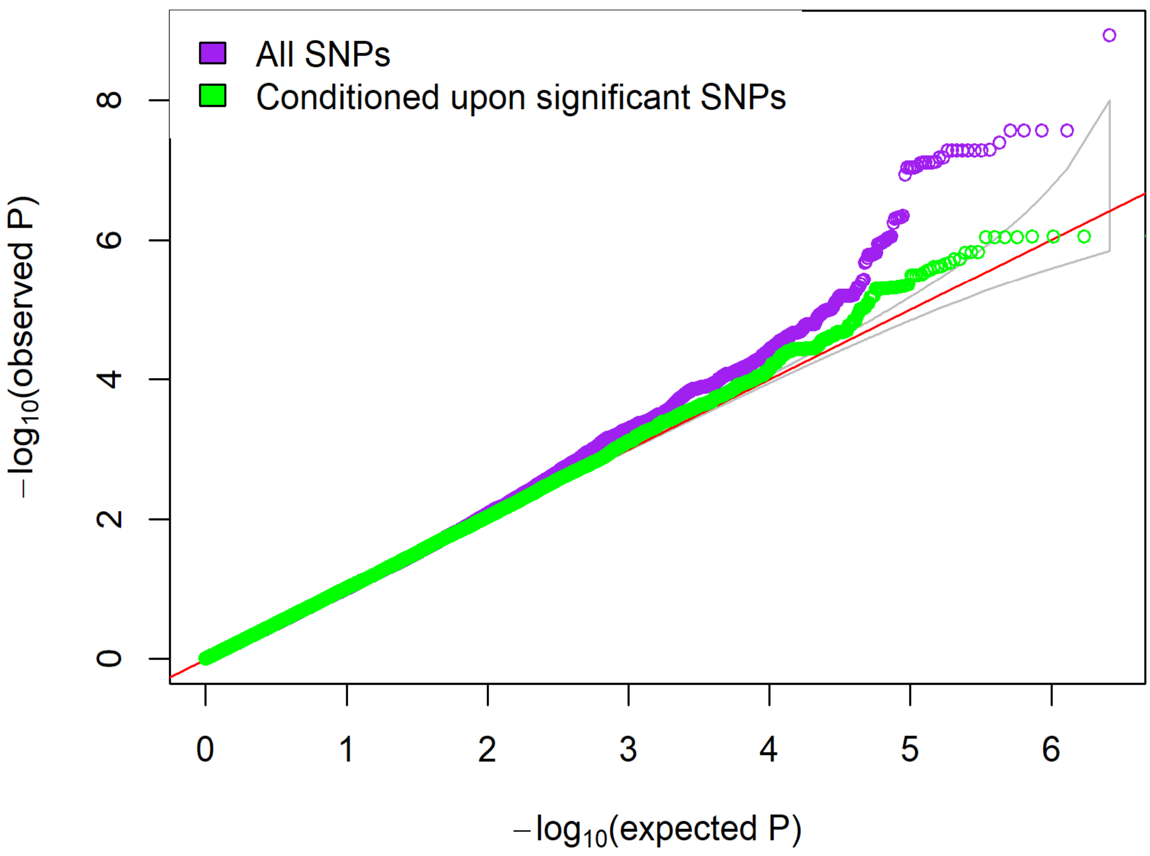

Figure S6B: Quantile-Quantile plot for conditional analysis for change in central macular thickness conditioning upon the genome-wide significant lead SNPs (rs78466540, rs11614480). The plot shows the expected −log10 p-values under the null hypothesis on the x-axis, and the observed −log10 p-values on the y axis adjusting for covariates Age, HbA1c, duration of diabetic retinopathy, injection type, central macular thickness, and the first three principal components. The red line represents x=y, and the grey area represents the 95% confidence interval. Purple dots represent plot when all SNPs were included (λ = 1.000) the green dots represent plot after conditioning by the significant SNPs (λ = 1.000).

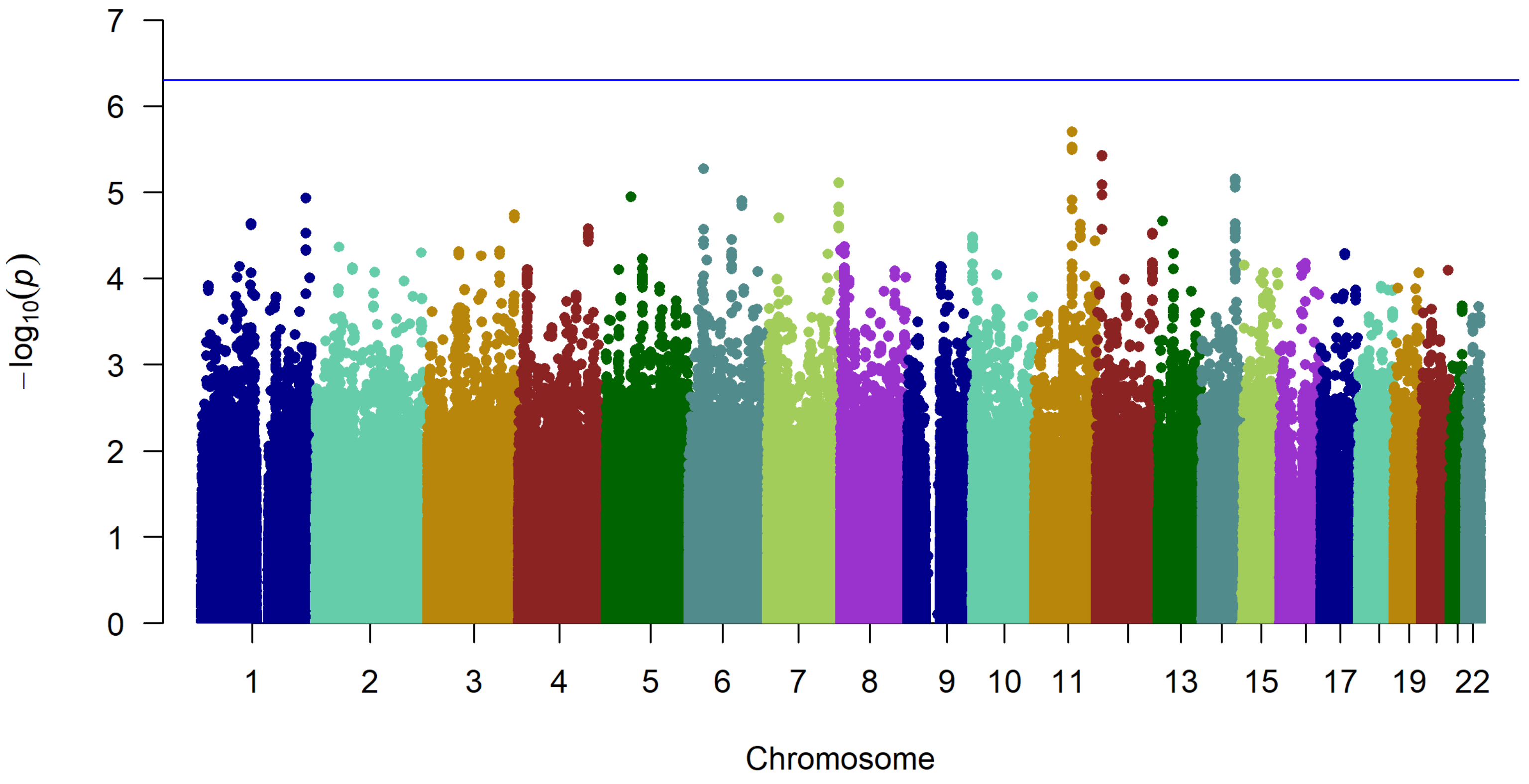

Figure S7A: Manhattan plot for conditional analysis for change in best-corrected visual acuity conditioning upon the genome-wide significant lead SNPs (rs148980760, rs187876551, rs57801753, and rs118074968). The x-axis represents chromosomal position, and the y-axis represents the −log10 (P-value) of association for each SNP tested. The blue horizontal line corresponds to the threshold for genome-wide suggestive association (P≤5E-07).

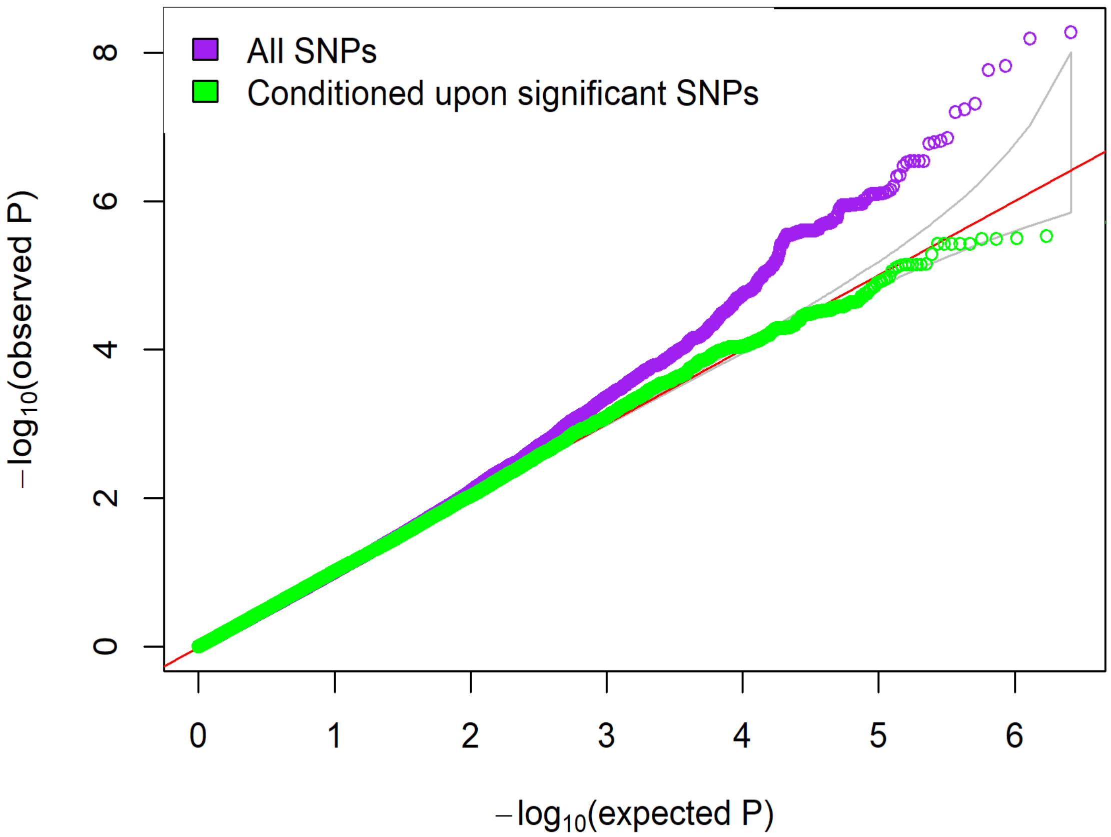

Figure S7B: Quantile-Quantile plot for conditional analysis for change in best-corrected visual acuity conditioning upon the genome-wide significant lead SNPs (rs148980760, rs187876551, rs57801753, and rs118074968). The plot shows the expected −log10 p-values under the null hypothesis on the x-axis, and the observed −log10 p-values on the y axis adjusting for covariates Age, HbA1c, duration of diabetic retinopathy, injection type, best-corrected visual acuity, and first three principal components. The red line represents x=y, and the grey area represents the 95% confidence interval. Purple dots represent plot when all SNPs were included (λ = 1.000) the green dots represent plot after conditioning by the significant SNPs (λ = 1.000).

Table S2: Baseline and clinical characteristics between responders and non-responders stratified by different categories

≥5 ETDRS letters criteria	Responders (N=114)	Non-responder (N=106)	*P* value
Individual related			
Baseline BCVA (ETDRS letters)	61.25 (14.01)	67.48 (14.42)	**0.001**
Baseline CMT (microns)	383.11 (90.28)	377.83 (117.51)	0.708
Age (years)	65.73 (12.68)	67.02 (11.59)	0.433
Male, N (%)	84 (73.7)	67 (63.2)	0.094
BMI (kg/m^2)	33.66 (7.66)	33.87 (8.37)	0.841
T2, N (%)	96 (84.2)	85 (80.2)	0.435
DM duration (years)	21.79 (10.55)	23.75 (9.65)	0.152
HbA1c (g/dl)	8.42 (1.63)	8.34 (1.63)	0.737
HTN:Yes, N (%)	99 (86.8)	89 (84.0)	0.545
Nephropathy: Yes, N (%)	62 (54.4)	58 (54.7)	0.961
Hyperlipidemia: Yes, N (%)	102 (89.5)	96 (90.6)	0.787
Smoker: Yes, N (%)	58 (50.9)	53(50.0)	0.897
Eye related			
Laterality: RE, N (%)	62 (54.40)	51 (48.10)	0.352
Lens: Phakic, N (%)	77(67.50)	71 (67.00)	0.958
DR duration (years)	7.49 (3.95)	8.68 (4.44)	0.047
DR severity: N (%)			0.813
Mild	1 (0.90)	0(0.00)	
Moderate	32(28.10)	35 (33.00)	
Severe	18 (15.80)	17 (16.00)	
PDR	39 (34.20)	33 (31.10)	
PRP: Yes, N (%)	48 (42.10)	42(39.60)	0.708
Injection number	8.05 (2.84)	7.99 (3.31)	0.896
Injection type: N (%)			0.538
Bevacizumab	61 (53.50)	65 (61.30)	
Ranibizumab	20 (17.50)	17 (16.00)	
Aflibercept	16 (14.00)	9 (8.50)	
Mixed	17 (14.90)	15 (14.20)	
**≥15 ETDRS letters criteria**	**Responders (N=31)**	**Non-responder (N=189)**	***P* value**
Individual related			
Baseline BCVA (ETDRS letters)	50.23 (15.37)	66.56 (13.04)	**< 0.001**
Baseline CMT (microns)	428.29 (111.13)	372.74 (101.05)	**0.006**
Age (years)	65.90 (11.39)	66.42 (12.31)	0.826
Male, N (%)	21 (67.7)	130 (68.8)	0.908
BMI (kg/m^2)	36.34 (9.31)	33.34 (7.71)	0.052

T2, N (%)	28 (98.3)	153 (81.0)	0.205
DM duration (years)	22.39 (12.77)	22.79 (9.70)	0.837
HbA1c (g/dl)	8.65 (1.51)	8.34 (1.64)	0.323
HTN:Yes, N (%)	26 (83.9)	162 (85.7)	0.787
Nephropathy: Yes, N (%)	17 (54.8)	103 (54.5)	0.972
Hyperlipidemia: Yes, N (%)	29 (93.5)	169 (89.4)	0.477
Smoker: Yes, N (%)	18 (58.1)	93(49.2)	0.361
Eye related			
Laterality: RE, N (%)	17 (54.80)	96 (50.80)	0.676
Lens: Phakic, N (%)	22 (71.00)	119 (63.00)	0.457
DR duration (years)	7.49 (3.95)	8.68 (4.44)	0.047
DR severity: N (%)			0.286
Mild	2 (6.50)	44(23.30)	
Moderate	11 (35.50)	56 (29.60)	
Severe	7 (22.60)	28 (14.80)	
PDR	11 (35.50)	61 (32.30)	
PRP: Yes, N (%)	12 (38.70)	78 (41.30)	0.788
Injection number	8.52 (3.15)	7.94 (3.06)	0.331
Injection type: N (%)			0.440
Bevacizumab	18 (58.10)	108 (57.10)	
Ranibizumab	4 (12.90)	33 (17.50)	
Aflibercept	2 (6.5)	23 (12.20)	
Mixed	7 (22.60)	25 (13.20)	
**Non-Responders≥ 5 ETDRS loss**	**Responders (N=178)**	**Non-responder (N=42)**	***P* value**
Individual related			
Baseline BCVA (ETDRS letters)	63.30 (14.82)	68.29 (12.52)	**0.045**
Baseline CMT (microns)	385.65 (104.95)	359.05 (98.62)	0.137
Age (years)	66.06 (12.40)	67.57 (11.15)	0.471
Male, N (%)	125 (70.20)	26 (61.90)	0.296
BMI (kg/m^2)	33.96 (8.29)	32.90 (6.64)	0.442
T2, N (%)	149 (83.70)	32 (76.20)	0.251
DM duration (years)	22.49 (10.16)	23.76 (10.21)	0.468
HbA1c (g/dl)	8.41 (1.65)	8.24 (1.54)	0.553
HTN:Yes, N (%)	154 (86.50)	34 (81.00)	0.358
Nephropathy: Yes, N (%)	100 (56.2)	20 (47.6)	0.316
Hyperlipidemia: Yes, N (%)	160 (89.90)	38 (90.50)	0.909
Smoker: Yes, N (%)	91 (51.1)	20(47.6)	0.683
Eye related			
Laterality: RE, N (%)	90 (50.60)	23 (54.80)	0.624
Lens: Phakic, N (%)	116 (65.20)	25 (59.50)	0.698
DR duration (years)	7.65 (4.31)	9.33 (4.04)	**0.022**
DR severity: N (%)			0.355

Mild	41 (23.10)	5 (11.90)	
Moderate	50 (28.10)	17 (40.50)	
Severe	27 (15.20)	8 (19.00)	
PDR	60 (33.70)	12 (28.60)	
PRP: Yes, N (%)	73 (41.00)	17 (40.50)	0.949
Injection number	8.13 (2.96)	7.57 (3.47)	0.295
Injection type: N (%)			0.272
Bevacizumab	99 (55.60)	27 (64.30)	
Ranibizumab	32 (18.00)	5 (11.90)	
Aflibercept	23 (12.90)	2 (4.80)	
Mixed	24 (13.50)	8 (19.00)	
**≥10% CMT reduction criteria**	**Responders (N=125)**	**Non-responder (N=95)**	
Individual related			
Baseline BCVA (ETDRS letters)	62.89 (16.32)	66.05 (11.58)	0.110
Baseline CMT (microns)	421.28 (113.08)	327.00 (57.15)	**<0.001**
Age (years)	67.05 (12.43)	65.43 (11.79)	0.330
Male, N (%)	21 (67.70)	130 (68.80)	0.598
BMI (kg/m^2)	32.91 (7.98)	34.88 (7.92)	0.070
T2, N (%)	105 (84.00)	76 (80.00)	0.442
DM duration (years)	22.06 (10.10)	23.62 (10.22)	0.261
HbA1c (g/dl)	8.11 (1.60)	8.74 (1.60)	**0.004**
HTN:Yes, N (%)	110 (88.00)	78 (82.10)	0.219
Nephropathy: Yes, N (%)	64 (51.20)	56 (58.90)	0.253
Hyperlipidemia: Yes, N (%)	114 (91.20)	84 (88.40)	0.496
Smoker: Yes, N (%)	60 (48.00)	51 (53.70)	0.404
Eye related			
Laterality: RE, N (%)	65 (52.00)	48 (50.50)	0.829
Lens: Phakic, N (%)	84 (67.20)	57 (60.00)	0.059
DR duration (years)	8.33 (4.31)	7.49 (4.28)	0.156
DR severity: N (%)			
Mild	32 (25.60)	14(14.80)	0.165
Moderate	37 (29.60)	30 (31.60)	
Severe	20 (16.00)	15 (15.80)	
PDR	36 (28.80)	36 (37.90)	
PRP: Yes, N (%)	47 (37.60)	43 (45.30)	
Injection number	8.06 (2.98)	7.97 (3.20)	0.834
Injection type: N (%)			
Bevacizumab	68 (54.40)	58 (61.10)	
Ranibizumab	25 (20.00)	12 (12.60)	
Aflibercept	17 (13.60)	8 (8.40)	
Mixed	15 (12.00)	17 (17.90)	

Abbreviations:BCVA=best corrected visual acuity; BMI=body mass index; CMT=central macular thickness; DM=diabetes mellitus; DR=diabetic retinopathy; ETDRS=early treatment diabetic retinopathy study;

HTN=hypertension; PDR=proliferative diabetic retinopathy; PRP=pan-retinal photocoagulation; RE=right eye
Data are presented as mean (SD).Chi-square test for categorical. Significant p-values are bolded

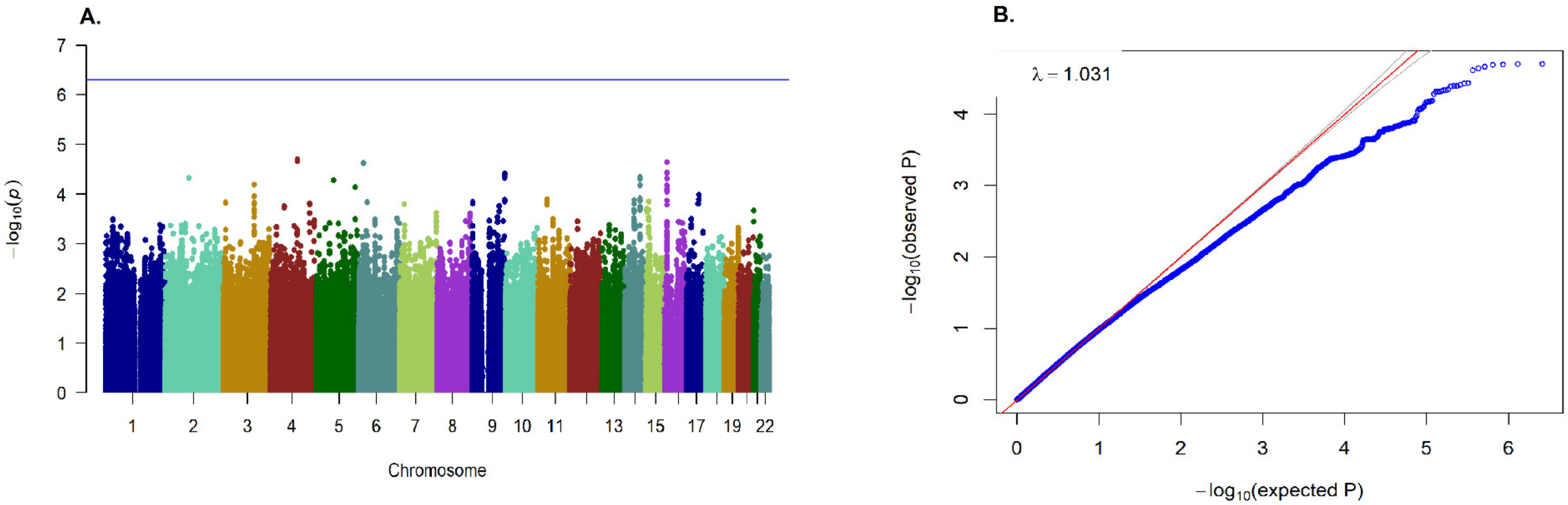

Figure S8: Logistic regression for functional response (Responder: ≥5 ETDRS letters improvement).

A: Manhattan plot for association analysis adjusting for baseline best-corrected visual acuity and the first three principal components. The x-axis represents chromosomal position, and the y-axis represents the −log10 (P-value) of association for each SNP tested. The blue horizontal line corresponds to genome-wide suggestive threshold (P≤5E-07).

B: Quantile-quantile plot shows the expected −log10 p-values under the null hypothesis on the x-axis, and the observed −log10 p-values on the y-axis adjusting for baseline best-corrected visual acuity and the first three principal components. The red line represents x=y, and the grey area represents the 95% confidence interval. The λ is a measure of the genomic inflation factor.

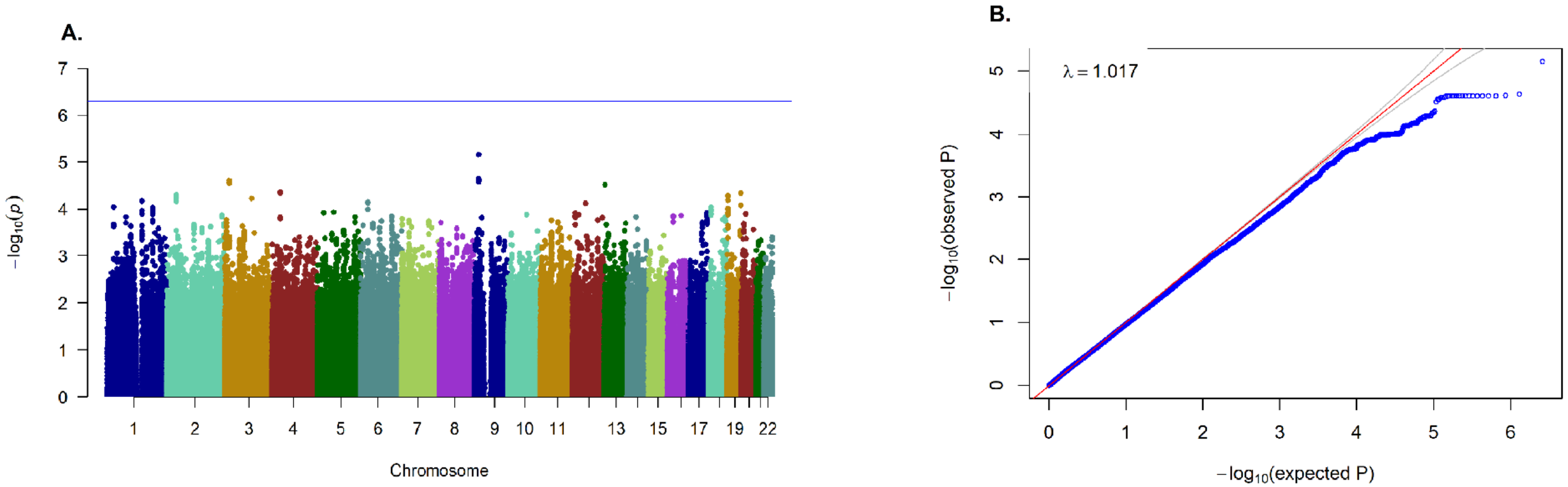

Figure S9: Logistic regression for functional response (Responder: ≥15 ETDRS letters improvement)

A: Manhattan plot for association analysis adjusting for baseline best-corrected visual acuity, baseline central macular thickness, and the first three principal components. The x-axis represents chromosomal position, and the y-axis represents the −log10 (P-value) of association for each SNP tested. The blue horizontal line corresponds to genome-wide suggestive threshold (P≤5E-07).

C: Quantile-quantile plot shows the expected −log10 p-values under the null hypothesis on the x-axis, and the observed −log10 p-values on the y-axis adjusting for baseline best-corrected visual acuity, baseline central macular thickness, and the first three principal components. The red line represents x=y, and the grey area represents the 95% confidence interval. The λ is a measure of the genomic inflation factor.

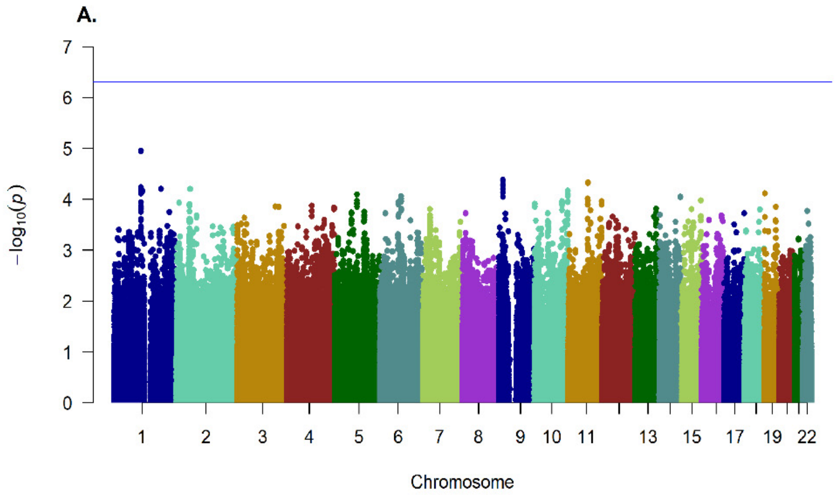

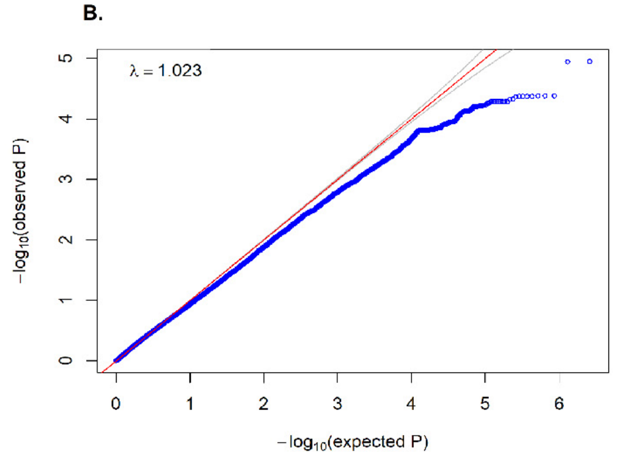

Figure S10: Logistic regression for functional response (Non-Responder: ≥5 ETDRS loss);

A: Manhattan plot of association analysis adjusting for baseline best-corrected visual acuity, duration of diabetic retinopathy, and the first three principal components. The x-axis represents chromosomal position, and the y-axis represents the −log10 (P-value) of association for each SNP tested. The blue horizontal line corresponds to genome-wide suggestive threshold (P≤5E-07).

B: Quantile-quantile plot shows the expected −log10 p-values under the null hypothesis on the x-axis, and the observed −log10 p-values on the y-axis adjusting for baseline best-corrected visual acuity, duration of diabetic retinopathy, and the first three principal components. The red line represents x=y, and the grey area represents the 95% confidence interval. The λ is a measure of the genomic inflation factor.

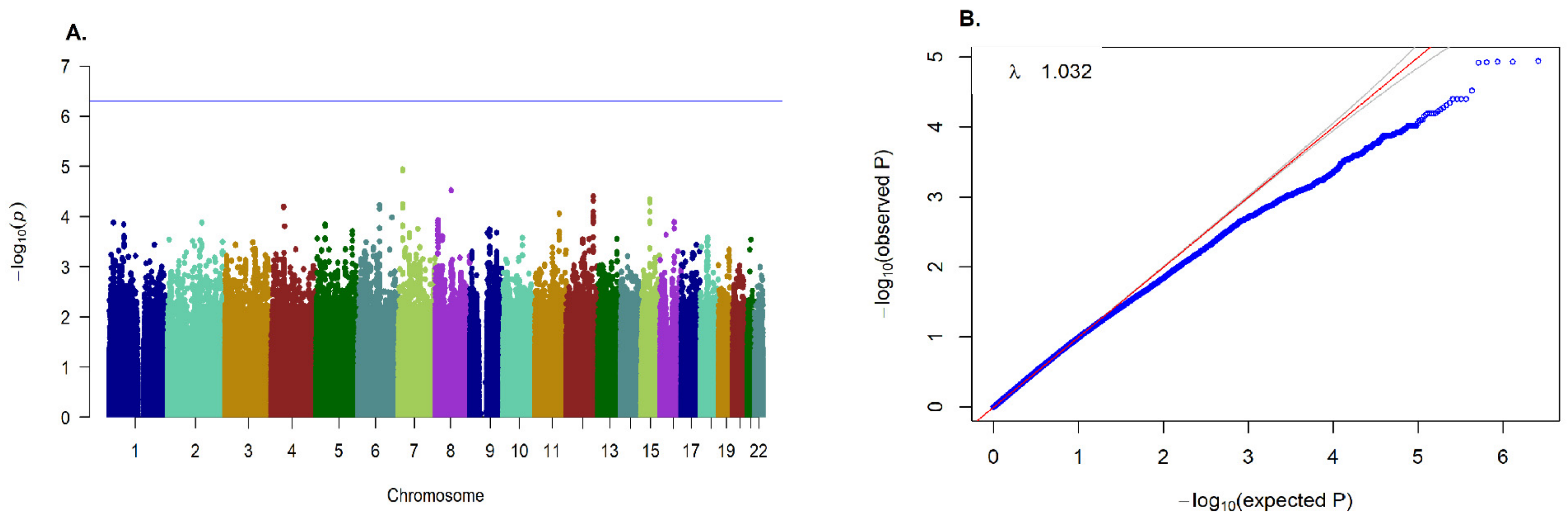

Figure S11: Logistic regression for anatomical response (Responder: ≥10% reduction in central macular thickness)

A: Manhattan plot of association analysis adjusting for baseline central macular thickness, HbA1c, and the first three principal components. The x-axis represents chromosomal position, and the y-axis represents the −log10 (P-value) of association for each SNP tested. The blue horizontal line corresponds to genome-wide suggestive threshold (P≤5E-07).

B: Quantile-quantile plot shows the expected −log10 p-values under the null hypothesis on the x-axis, and the observed −log10 p-values on the y-axis adjusting for baseline central macular thickness, HbA1c, and the first three principal components. The red line represents x=y, and the grey area represents the 95% confidence interval. The λ is a measure of the genomic inflation factor.

Table S3: Expression data (PLIER score) of the potential candidate genes for different eye tissues retrieved from the Ocular Tissue Database (OTDB).

	**Genes**	**Choroid retinal pigment epithelium (RPE)**	**Ciliary body**	**Cornea**	**Iris**	**Lens**	**Optic Nerve**	**Optic Nerve Head**	**Retina**	**Sclera**	**Trabecular Meshwork**
Significant loci	*CASC15*	-	-	-	-	-	-	-	-	-	-
	RP11-116D17.1	-	-	-	-	-	-	-	-	-	-
	NTM	36.1744	25.4854	21.2339	28.9124	33.5313	59.0734	44.1297	73.7647	36.1583	32.2057
	PGAM1P1	-	-	-	-	-	-	-	-	-	-
	RP11-744N12.3	-	-	-	-	-	-	-	-	-	-
	TBC1D32	-	-	-	-	-	-	-	-	-	-
Suggestive loci	*EPHA5*	18.9253	23.8659	20.1597	24.8548	23.7192	24.4528	23.3787	22.264	23.8881	13.8842
	DNASE1	40.2426	32.7204	35.0726	39.5583	32.8458	30.6663	29.7455	31.4745	30.5837	37.8598
	AP003733.1	-	-	-	-	-	-	-	-	-	-
	RP11-179A16.1	-	-	-	-	-	-	-	-	-	-
	AC069243.1	-	-	-	-	-	-	-	-	-	-
	COL4A1	50.7142	42.6072	24.592	39.6132	54.5327	26.2596	29.0696	30.377	30.395	46.7098

	JPH1	20.5319	17.1471	29.4043	15.0664	27.3079	13.2068	17.8659	14.6479	19.5787	19.3243
	RP6-91H8.2	-	-	-	-	-	-	-	-	-	-
eQTL	*RP11-116D17.3*	-	-	-	-	-	-	-	-	-	-
	GJA1	887.985	890.373	1378.98	1713.06	553.158	1193.29	1404.32	61.0931	379.516	566.688
Note: '-' indicates that the gene was not present in the OTDB.											
